# Stress induced experimental colitis

**DOI:** 10.1155/S0962935193000353

**Published:** 1993

**Authors:** T. A. Stein, L. Keegan, L.-J. Auguste, B. Bailey, L. Wise

**Affiliations:** Department of Surgery Long Island Jewish Medical Center The Long Island Campus for the Albert Einstein College of Medicine 270-05 76th Avenue New Hyde Park NY 11042 USA

## Abstract

Stress induces chemical changes in the central nervous system which alters the biochemistry and physiology of the digestive tract. The present study determines arachidonic acid oxidation and damage in the colon following stress. Ten rats were stressed by the cold-restraint method; ten were controls. Stress induced 0.5 ± 0.7 (S.D.) mucosal erosions whereas controls had none. Subepithelial hemorrhage and erosions occurred only in the proximal two-thirds of the colon. Prostaglandin E_2_ synthesis was increased after stress compared to the control (381 ± 130 vs. 1610 ± 372 ng/g/min). Leukotriene C_4_ synthesis also increased after stress (4217 ± 994 vs. 11300 ± 1662 ng/g/min). Synthesis of prostaglandin E_2_ increased (r = 0.9381) with leukotriene C_4_. The response of the colon to stress is less severe than that in the stomach and may be related to regional regulation of prostaglandin and leukotriene synthesis.


Mediators of Inflammation, 2, 243–246 (1993)

**PREPARATIONS of murine recombinant interferon (Mu-rIFN)-alpha, -beta and -gamma were assessed for their influence on *in vitro* growth of mast cells from normal mouse spleen cells (Sp C). Mast cell growth was inhibited by Mu-rIFNs when Sp C were exposed throughout the entire culture period to Mu-rIFNs. The most potent inhibitor of mast cell growth was Mu-rIFN-$\gamma$, followed by Mu-rIFN-$\beta$; Mu-rIFN-$\alpha$ had little effect. When added to IC-2 cells, clonal mast cell progenitor, both Mu-rIFN-$\beta$ and -$\gamma$ significantly inhibited proliferative response of the target cells. The suppressive effect of Mu-rIFNs on IC-2 cells was selectively abolished by monoclonal antibodies against Mu-rIFN-$\beta$ and -$\gamma$.**

**Key words:** Interferon, Mast cell, Mice

### Inhibitory effect of interferon-beta on mouse spleen-derived mast cells

**S. Konno,[1,CA] M. Adachi,[1] K. Asano,[2] K. Okamoto[2] and T. Takahashi[1]**

[1]First Department of Internal Medicine, and [2]Department of Medical Biology, School of Medicine, Showa University, Hatanodai, Shinagawa-ku, Tokyo 142, Japan

[CA] Corresponding Author

### Introduction

Interferons (IFNs) are well known to be a family of inducible proteins secreted by several types of cells in response to viruses and other stimuli. It is also generally accepted that IFNs can be divided into three different subclasses, IFN-$\alpha$, -$\beta$ and -$\gamma$ based on their structure, cellular origin and biological properties.

The effect of IFNs on cell proliferation was examined extensively by many investigators and IFN-$\alpha$ and -$\gamma$ have been shown to suppress the *in vitro* proliferation and/or differentiation of human pulripotent hemopoietic progenitor cells and of committed progenitor cells.[1–3] Recently, Nafzigar *et al.*,[4] using normal mouse bone marrow cells, examined the effect of IFN-$\gamma$ on mast cell growth and reported that IFN-$\gamma$ strongly inhibited their growth when bone marrow cells were exposed throughout the entire culture period to IFN-$\gamma$ which seemed to exert its inhibitory activity on mast cell progenitors. There is, however, no evidence for a direct effect of IFN-$\gamma$ on mast cell progenitors. Furthermore, no information is yet available concerning the effects of IFN-$\alpha$ and -$\beta$ on mast cell growth and proliferation.

The aim of the present study, therefore, is to evaluate the *in vitro* effects of IFN-$\alpha$ and -$\beta$ on mast cells. In addition, studies were performed to investigate whether an inhibitory activity of IFN-$\gamma$ on mast cell growth was due to a direct effect on progenitor cells.

### Materials and Methods

*Mice:* Male BALB/c mice, 7 weeks old, were purchased from Charles River Japan Inc., Atsugi, Japan.

*Cell line:* Clonal mast cell progenitor IC-2 cell line[5] was kindly supplied by Cell Bank, Riken Gene Bank, Ibaragi, Japan.

*IFN and monoclonal antibody to IFN:* Murine-recombinant IFN (Mu-rIFN)-$\alpha$ (Spe. Act.: $1.1 \times 10^6$ U/ml) and -$\beta$ (specific activity $1.1 \times 10^6$ U/ml) were purchased from Lee Biomolecular Research Lab., Inc., San Diego, CA. Mu-rIFN-$\gamma$ (specific activity $1.9 \times 10^6$ U/ml) was kindly provided by Toray (Kanagawa, Japan). To neutralize the activity of Mu-rIFNs, 25 $\mu$g of monoclonal antibodies to Mu-rIFN-$\beta$ (Lee Biomolecular Research Lab. Inc.) and to Mu-rIFN-$\gamma$ (Genzyme Corp., Cambridge, MA) were incubated with 100 $\mu$l of the appropriate Mu-rIFN preparations for 90 min at 37°C just before use.

*Induction of mast cells from normal mouse spleen cells:* Spleen cell suspension (Sp C) was prepared as described previously.[6] The cells were adjusted to $5 \times 10^6$ cells/ml or $10 \times 10^6$ cells/ml in RPMI-1640 medium (Flow Lab., Irvine, Scotland) supplemented with 10% foetal calf serum (Bocknek, Canada), 25 mM HEPES, 200 mM L-glutamine, 100 U/ml penicillin 100 $\mu$g/ml streptomycin, and 30% interleukin 3 culture supplement (Collaborative Biomedical products, Bedford, MA). The cells (1 ml/well) were

© 1993 Rapid Communications of Oxford Ltd incubated in 24-well culture plates (Nunc, Intermed, Denmark) at 37°C in a humidified atmosphere with 5% $CO_2$.

*IFN treatment:* Mu-rIFNs were added to cultured cells at seeding and then every 4 days, when the culture medium was replaced with fresh medium. To study the effects of Mu-rIFNs at different stages of mast cell growth, another series of experiments was carried out, in which Mu-rIFNs were added at seeding or 10 days after seeding.

*Cell counts:* Every 4 days, the growth of cells was measured by counting cells with a hemocytometer. Cell viability was determined by trypan blue exclusion. Mast cells were counted by a staining method with alcian blue specific for counting basophils and mast cells.[7]

*Cell proliferation:* IC-2 cells at a concentration of $2 \times 10^5$ cells/ml were introduced into each well of 96-well flat-bottomed culture plates (Nunc, Intermed) that contained Mu-rIFNs in a final volume of 0.2 ml. The plate was maintained at 37°C in a humidified atmosphere with 5% $CO_2$. After 40 h, 1.0 $\mu$Ci of $^3$H-thymidine (specific activity 25 Ci/mM, Amersham International Plc, Buckinghamshire, UK) was added and the plate was maintained for a further 8 h. Cell proliferation was assessed by determination of the rate of incorporation of $^3$H-thymidine into DNA as described previously.[6]

*Statistical analysis:* Data from control and experimental groups were compared by Student's *t*-test.

### Results

*Influence of IFNs on mast cell growth:* To examine effects of Mu-rIFNs on mast cell induction, $10 \times 10^6$ Sp C were cultured in the presence of either IFN-$\alpha$, -$\beta$ or -$\gamma$ (100 U/ml each), and the numbers of viable cells and mast cells were counted. As the culture time went on, viable cell numbers decreased gradually in the culture (Fig. 1A). When Sp C were cultured without Mu-rIFNs (Control), the mast cell numbers were gradually increased, peaked on the sixteenth day (Fig. 1B). In contrast to the results obtained in cultures exposed to Mu-rIFN-$\alpha$, a statistically significant suppression of mast cell growth was observed when Sp C were exposed throughout the entire culture period to Mu-rIFN-$\beta$ and -$\gamma$ (Fig. 1B).

The second experiments were carried out to examine whether mast cell growth inhibition by Mu-rIFN-$\beta$ and -$\gamma$ was associated with concentration of Mu-rIFNs added to cultures. To accomplish this, $5 \times 10^6$ Sp C were cultured in the presence of four different doses of Mu-rIFNs and the number of mast cells was counted. As shown in Fig. 2, dose-dependent suppression was observed: 50 and 100 U/ml of Mu-rIFN-$\beta$ failed to suppress mast cell growth but 500 and 1000 U/ml strongly suppressed them. The data in Fig. 2 also indicated that the suppression exerted by Mu-rIFN-$\gamma$ was stronger than that by Mu-rIFN-$\beta$.

The third experiments were designed to examine the effects of Mu-rIFNs in different stages of mast cells. As shown in Fig. 3, addition of Mu-rIFN-$\beta$ and -$\gamma$ (1000 U/ml each) at seeding of $5 \times 10^6$ Sp C significantly inhibited mast cell

FIG. 1. Influence of interferons on viable cells and mast cell induction in cultured murine splenocytes. Mu-rIFNs (1000 U/ml) were added at the time of seeding spleen cells ($10 \times 10^6$ cells/ml) and then every 4 days, when the culture medium was replaced with the fresh medium. Results are expressed as the mean ± S.E. of two different experiments.

FIG. 2. Effects of murine-recombinant interferons (Mu-rIFNs) on mast cell growth. Mu-rIFNs were added at the time of seeding spleen cells ($5 \times 10^6$ cells/ml) and then every 4 days, when the culture medium was replaced with a fresh medium. Mast cell growth was evaluated 16 days after culture. Results are expressed as the mean ± S.E. of two different experiments. Asterisks indicate $p < 0.05$ versus control (None).

FIG. 3. Effects of adding murine-recombinant interferons (Mu-rIFNs) at different times after seeding spleen cells. Mu-rIFNs were added to the culture medium on days 0 and 10, and mast cell growth was evaluated on day 16. The values are the mean ± S.E. of two different experiments. Asterisks indicate $p < 0.05$ versus control.

growth. However, no such inhibition was observed when Mu-rIFNs were added to the cells after 10 days of culture.

*Influence of Mu-rIFNs on mast cell progenitors:* The final experiments were carried out to examine whether Mu-rIFN-β and -γ exerted their inhibitory effects described above by acting directly on mast cell precursors. For this purpose, IC-2 cells, mast cell progenitors were cultured in the presence of Mu-rIFNs and cell proliferation was examined. As shown in Table 1, addition of Mu-rIFN-β and -γ significantly inhibited IC-2 cell proliferation in dose

**Table 1.** Effect of interferons (IFN) on the proliferation of IC-2 cell line

| Dose of IFN (U/ml) | Proliferation (cpm ± S.E.) | |
|---|---|---|
| | IFN-β | IFN-γ |
| None (control) | 272 452.0 ± 4 401.5 | 252 742.0 ± 10 430.1 |
| 50 | 262 153.7 ± 21 504.1 | 254 093.3 ± 2 218.7 |
| 100 | 221 744.0 ± 7 053.4 | 112 901.7 ± 17 508.0* |
| 500 | 206 167.1 ± 9 350.1* | 92 088.1 ± 4 032.5* |
| 1000 | 130 673.4 ± 38 264.0* | 47 678.3 ± 3 504.4* |

Clonal mast cell precursors, IC-2 cells, at a concentration of $2 \times 10^5$ cells/ml were cultured with $^3$H-thymidine in a culture plate containing Mu-rIFNs. Asterisks indicate $p < 0.05$ versus control.

**Table 2.** Specific inactivation of the suppressive influence of murine-recombinant interferons (Mu-rIFNs)-β and -γ on IC-2 cell proliferation by pre-incubation of the interferons with their respective antibodies

| | $^3$H-thymidine uptake (cpm ± S.E.) |
|---|---|
| Control (medium alone) | 276 728.3 ± 8 228.7 |
| Mu-rIFN-β ($10^3$ U/ml) | 76 454.3 ± 6 544.4* |
| Mu-rIFN-β + anti-IFN-beta | 279 116.3 ± 4 370.3 |
| Mu-rIFN-γ ($10^3$ U/ml) | 41 995.0 ± 3 011.1* |
| Mu-rIFN-γ + anti-IFN-gamma | 214 557.3 ± 7 428.1 |

Preparations of Mu-rIFNs were preincubated for 90 min at 37°C with or without $10^4$ neutralizing units of monoclonal antibodies to Mu-rIFN-β or Mu-rIFN-γ. Asterisks indicate $p < 0.05$ versus control.

dependent manner. These inhibitory actions were completely abolished when Mu-rIFNs were pre-incubated with monoclonal antibodies suitable to the appropriate IFN type (Table 2).

### Discussion

The results of this study demonstrated that: (1) both rIFN-β and -γ, but not -α, are potent inhibitors of murine mast cell proliferation; (2) rIFN-β and -γ exert their inhibitory activity on mast cell progenitors, but not on mature mast cells. These conclusions are supported by the following observations: firstly, when Sp C were exposed throughout the entire culture period to rIFN-β and -γ, a statistically significant and dose-dependent suppression of mast cell growth was observed, whereas no such inhibition was observed when Mu-rIFN-α was added to the cultures at seeding of Sp C. Secondly, addition of rIFN-β and -γ to the culture medium at seeding strongly inhibited mast cell growth. However, addition of rIFNs 10 days after seeding, when differentiation of precursors and proliferation of immature mast cells are progressed, did not affect mast cell growth, as assessed by mast cell number observed on day 16. Finally, proliferation of IC-2 cells, clonal mast cell progenitors, was strongly inhibited when the cells were cultured *in vitro* with rIFN-β and -γ. This inhibition was completely neutralized when the IFNs were pre-incubated with a monoclonal antibody directed to the appropriate IFN type.

As far as we know, the present experiments are the first to demonstrate that IFN-γ exerts its antiproliferative effect on mast cells by acting directly on their precursors and to show that IFN-β, but not IFN-α has biological effects on mast cells similar to those of IFN-γ.

IFN-β could exert its anti-proliferative effect by acting directly on mast cell precursors and various mechanisms can be envisaged. IFN-β might act on Ia antigen and result in inhibition of mast cell growth. Indeed, under certain conditions, Ia antigen was shown to be involved in the negative regulation of cell growth.[8,9] Alternatively, down-regulation of oncogene expression, demonstrated by the addition of IFNs to *in vitro* experimental models,[10] might also explain the anti-proliferative activity of IFN-$\beta$ on mast cells. The possibility that other cells present in early cultures could be involved in the mediation of inhibitory effect of IFN-$\beta$ cannot be excluded, since a role of accessory cells such as lymphocytes and monocytes was reported in the suppression of normal erythropoiesis by IFN-$\gamma$.[11] It is well known that IFNs exert their pleiotropic effects by interacting with specific receptors on the surface membrane. Nafzigar *et al.*[4] reported that IFN-$\gamma$ inhibits *in vitro* mast cell growth by formation of IFN-$\gamma$-receptor complex, suggesting that IFN-$\beta$ binds to specific receptor for IFN-$\beta$ differed from that for IFN-$\gamma$[12,13] on mast cell precursors and result in inhibition of mast cell growth. Anyway, further experimentations are needed to clarify the precise mechanisms by which IFN-$\beta$ inhibits mast cell growth.

IFNs have been employed successfully in the treatment of some infectious diseases and cancers.[14] So far, no clinical applications have been made of their potent immunoregulatory effects, although the inhibitory effect of IFNs on the mycotic disease induced by *Aspergilus fumigatus*[15] and on lepromatous leprosy[16] may depend on the activation of macrophages and T-cells, respectively. Mast cells are now considered to play a pivotal role not only in allergic reactions but also in many pathological immune responses.[17,18] It is also well known that infiltration of mast cells and the presence of mast cell-derived soluble mediators in the presence of asthma and atopic allergy are well correlated with the severity of the diseases. From these reports, whatever the mechanisms involved, our *in vitro* results suggest that IFN-$\beta$ and -$\gamma$ could act *in vivo* as inhibitory factors of mast cell proliferation and that these lymphokines could be of interest in the treatment of asthma and atopic allergy.

### References

1. Broxmeyer HE, Lu L, Platzer E, Feit C, Juliano L, Rubin BY. Comparative analysis of the influences of human $\gamma$, $\alpha$ and $\beta$ interferons on human multipotential (CFU-GEMM), erythroid (BFU-E) and granulocyte-macrophage (CFU-GM) progenitor cells. *J Immunol* 1983; **131:** 1300–1305.
2. Ganser A, Carlo SC, Greher J, Volkers B, Hoelzer D. Effect of recombinant interferons alpha and gamma on human bone marrow-derived megakaryocytic progenitor cells. *Blood* 1987; **70:** 1173–1179.
3. Raefsky EL, Platanias LC, Zoumbos NC, Young NS. Studies of interferon as a regulator of hematopoietic cell proliferation. *J Immunol* 1985; **135:** 2507–2512.
4. Nafziger J, Arock M, Guillosson JJ, Wietzerbin J. Specific high-affinity receptors for interferon-$\gamma$ on mouse bone marrow-derived mast cells: inhibitory effect of interferon-$\gamma$ on mast cell precursors. *Eur J Immunol* 1990; **20:** 113–117.
5. Koyasu S, Yodoi J, Nikaido T, *et al.* Expression of interleukin 2 receptors on interleukin 3-dependent cell lines. *J Immunol* 1986; **136:** 984–987.
6. Konno S, Adachi M, Asano K, Kawazoe T, Okamoto K, Takahashi T. Influence of roxithromycin on cell-mediated immune responses. *Life Sci* 1992; **51:** PL107–PL112.
7. Gilbert H, Sornstein L. Basophil counting with a new staining method using alcian blue. *Blood* 1975; **46:** 279–286.
8. Pelus LM, Saletan S, Silver RT, Moore MA. Expression of Ia-antigens on normal and chronic myeloid leukemic human granulocyte macrophage colony-forming cells (CFU-GM) is associated with the regulation of cell proliferation by prostaglandin E. *Blood* 1982; **59:** 284–292.
9. Wong GH, Clark LI, Hamilton JA, Schrader JW. P cell stimulating factor and glicocorticoids oppose the action of interferon-$\gamma$ in inducing Ia antigens on T-dependent mast cells (P cells). *J Immunol* 1984; **133:** 2043–2050.
10. Kimchi A, Resnitzky D, Ber R, Gat G. Recessive genetic deregulation abrogates c-*myc* suppression by interferon and is implicated in oncogenesis. *Mol Cell Biol* 1988; **8:** 2828–2836.
11. Mamus SW, Beck SS, Zanjani ED. Suppression of normal human erythropoiesis by gamma interferon *in vitro*. Role of monocytes and T-lymphocytes. *J Clin Invest* 1985; **75:** 1496–1503.
12. Orchansky P, Novick D, Fischer DG, Rubinstein M. Type I and Type II interferon receptors. *J Interferon Res* 1984; **4:** 275–282.
13. Celada A. The interferon gamma receptor. *Lymphokine Res* 1988; **7:** 61–73.
14. Friedman RM. Interferons. In: Oppenheim JJ, Schevach EM, eds. *Immunophysiology*. New York: Oxford University Press, 1990; 194–209.
15. Maheshwari RK, Tandon RN, Rhodes-Feuillette A, Mahou G, Badillet G, Friedman RM. Interferon inhibits *Aspergillus fumigatus* growth in mice: an activity against an extracellular infection. *J Interferon Res* 1987; **8:** 35–44.
16. Nathan CF, Kaplan G, Levis WR, *et al.* Local and systemic effects of intradermal recombinant interferon in patients with lepromatous leprosy. *N Engl J Med* 1986; **315:** 6–14.
17. Stevens RL, Austen KF. Recent advances in the cellular and molecular biology of mast cells. *Immunol Today* 1989; **10:** 381–386.
18. Galli SJ. New insights into 'The riddle of the mast cells': microenvironmental regulation of mast cell development and phenotypic heterogeneity. *Lab Invest* 1990; **62:** 5–33.

ACKNOWLEDGEMENTS. We thank Dr T. Ohno, Cell Bank, Riken Gene Bank, for providing the IC-2 cell line. We also acknowledge Dr Kajita (Toray Industries, Inc. Basic Research Laboratories) for a kind gift of recombinant murine interferon-$\gamma$.

**Received 3 February 1993; accepted in revised form 7 April 1993**

## References

[PDF_00379] Celada A. (1988). The interferon gamma receptor.. Lymphokine Res.

[PDF_00390] Galli S. J. (1990). New insights into "the riddle of the mast cells": microenvironmental regulation of mast cell development and phenotypic heterogeneity.. Lab Invest.

[PDF_00347] Ganser A., Carlo-Stella C., Greher J., Völkers B., Hoelzer D. (1987). Effect of recombinant interferons alpha and gamma on human bone marrow-derived megakaryocytic progenitor cells.. Blood.

[PDF_00362] Gilbert H. S., Ornstein L. (1975). Basophil counting with a new staining method using alcian blue.. Blood.

[PDF_00371] Kimchi A., Resnitzky D., Ber R., Gat G. (1988). Recessive genetic deregulation abrogates c-myc suppression by interferon and is implicated in oncogenesis.. Mol Cell Biol.

[PDF_00359] Konno S., Adachi M., Asano K., Kawazoe T., Okamoto K., Takahashi T. (1992). Influences of roxithromycin on cell-mediated immune responses.. Life Sci.

[PDF_00357] Koyasu S., Yodoi J., Nikaido T., Tagaya Y., Taniguchi Y., Honjo T., Yahara I. (1986). Expression of interleukin 2 receptors on interleukin 3-dependent cell lines.. J Immunol.

[PDF_00382] Maheshwari R. K., Tandon R. N., Feuillette A. R., Mahouy G., Badillet G., Friedman R. M. (1988). Interferon inhibits Aspergillus fumigatus growth in mice: an activity against an extracellular infection.. J Interferon Res.

[PDF_00374] Mamus S. W., Beck-Schroeder S., Zanjani E. D. (1985). Suppression of normal human erythropoiesis by gamma interferon in vitro. Role of monocytes and T lymphocytes.. J Clin Invest.

[PDF_00353] Nafziger J., Arock M., Guillosson J. J., Wietzerbin J. (1990). Specific high-affinity receptors for interferon-gamma on mouse bone marrow-derived mast cells: inhibitory effect of interferon-gamma on mast cell precursors.. Eur J Immunol.

[PDF_00385] Nathan C. F., Kaplan G., Levis W. R., Nusrat A., Witmer M. D., Sherwin S. A., Job C. K., Horowitz C. R., Steinman R. M., Cohn Z. A. (1986). Local and systemic effects of intradermal recombinant interferon-gamma in patients with lepromatous leprosy.. N Engl J Med.

[PDF_00377] Orchansky P., Novick D., Fischer D. G., Rubinstein M. (1984). Type I and Type II interferon receptors.. J Interferon Res.

[PDF_00364] Pelus L. M., Saletan S., Silver R. T., Moore M. A. (1982). Expression of Ia-antigens on normal and chronic myeloid leukemic human granulocyte-macrophage colony-forming cells (CFU-GM) is associated with the regulation of cell proliferation by prostaglandin E.. Blood.

[PDF_00350] Raefsky E. L., Platanias L. C., Zoumbos N. C., Young N. S. (1985). Studies of interferon as a regulator of hematopoietic cell proliferation.. J Immunol.

[PDF_00388] Stevens R. L., Austen K. F. (1989). Recent advances in the cellular and molecular biology of mast cells.. Immunol Today.

